# Combinations of siRNAs against La Autoantigen with NS5B or hVAP-A Have Additive Effect on Inhibition of HCV Replication

**DOI:** 10.1155/2016/9671031

**Published:** 2016-06-29

**Authors:** Anirban Mandal, Krishna Kumar Ganta, Binay Chaubey

**Affiliations:** ^1^Centre for Advance Studies, Department of Botany, University of Calcutta, 35 Ballygunge Circular Road, Kolkata 700019, India; ^2^Laboratory of Recombinant Vaccines, Intercollegiate Faculty of Biotechnology, UG and MUG, Abrahama 58 Street, 80-307 Gdańsk, Poland

## Abstract

Hepatitis C virus is major cause of chronic liver diseases such as chronic hepatitis, liver cirrhosis, and hepatocellular carcinoma. Presently available direct-acting antiviral drugs have improved success rate; however, high cost limits their utilization, especially in developing countries like India. In the present study, we evaluated anti-HCV potential of several siRNAs targeted against the HCV RNA-dependent RNA polymerase NS5B and cellular factors, La autoantigen, PSMA7, and human VAMP-associated protein to intercept different steps of viral life cycle. The target genes were downregulated individually as well as in combinations and their impact on viral replication was evaluated. Individual downregulation of La autoantigen, PSMA7, hVAP-A, and NS5B resulted in inhibition of HCV replication by about 67.2%, 50.7%, 39%, and 52%, respectively. However, antiviral effect was more pronounced when multiple genes were downregulated simultaneously. Combinations of siRNAs against La autoantigen with NS5B or hVAP-A resulted in greater inhibition in HCV replication. Our findings indicate that siRNA is a potential therapeutic tool for inhibiting HCV replication and simultaneously targeting multiple viral steps with the combination of siRNAs is more effective than silencing a single target.

## 1. Introduction

HCV is an enveloped, single-stranded positive sense, RNA virus belonging to Flaviviridae family that causes acute and chronic hepatitis in humans. Persistent HCV infection often leads to cirrhosis and hepatocellular carcinoma [1]. Currently, 130–150 million people are chronically infected with HCV and ~0.5 million people die every year due to HCV related liver diseases [2]. World Health Organization (WHO) has recognized hepatitis C as a global health problem [3]. Till 2011, the mainstay of the HCV therapy has been a combination of pegylated interferon alfa (PEG-IFN *α*) in combination with ribavirin [4, 5]. The addition of telaprevir and boceprevir (NS3/4A protease inhibitors) to this arsenal improved success rate, but rapid development of drug resistance, severe side effects, and low spectrum activity proved to be barriers to treatment, and therefore it is no longer used after interferon-free regimens became widely available [6–9]. HCV treatment has been further improved with the recent introduction of HCV NS5B inhibitor sofosbuvir and NS5A inhibitor ledipasvir [10]. In early 2016 second-generation NS3/4A protease inhibitor grazoprevir and second-generation NS5A inhibitor elbasvir were approved for the treatment of genotypes 1 and 4 infected patients [11]. However, due to the poor replicative fidelity of HCV, frequent emergences of drug-resistant strains are inevitable. Furthermore, high cost and genotype dependency of these drugs often lead to treatment noncompliance [12, 13]. This necessitates a continuing effort to design and develop additional agents to combat HCV.

RNA interference (RNAi) is a gene silencing phenomenon during which small dsRNA molecules induce sequence-specific degradation of homologous endogenous mRNA [14, 15]. In general, HCV is highly susceptible to RNAi because HCV genome is a (+) single-stranded RNA which functions as both messenger RNA and template for replication in the cytoplasm of hepatocyte [16]. Silencing of HCV RNA could stop its replication and propagation. Therefore, utilization of this technology holds tremendous potential as a therapeutic tool against HCV. Previous reports have shown that silencing of cellular or viral determinants could inhibit HCV entry and replication [17–23]. siRNAs targeted against the core and NS4B regions specifically decreased viral load in a dose-dependent manner [24]. siRNAs against NS5B and NS3 regions inhibit HCV replication and protein expression without any IFN response [25]. Alternatively, many studies have also been focused on the cellular factors that directly or indirectly interact with the different regions of the viral genome and play a vital role in viral life cycle [26–29]. However, monotherapy with siRNA has led to the emergence of viral escape mutants through point mutation within the siRNA target regions [30, 31]. Therefore, combinations of siRNAs comprising multiple viral and/or cellular targets have also been evaluated. Few recent reports showed that combinations of siRNAs targeted against different cellular or viral factors have synergistic anti-HCV effects [32–36]. Most of these studies were restricted to either viral entry or the viral genome. However, different stages in HCV life cycle, such as viral entry, viral genome translation, replication complex formation and replication, and virion assembly and release, represent a broader spectrum of molecular events that may serve as potential targets against HCV. Therefore, we propose cocktail of siRNAs targeting different viral and cellular factors which may intercept various crucial steps in the viral life cycle and may enforce a strong synergistic antiviral effect.

In the present study, gene specific siRNAs were designed against cellular factors, La autoantigen, PSMA7, hVAP-A, and viral factor HCV NS5B and screened for HCV inhibition individually as well as in combinations. These cellular factors and viral protein NS5B play crucial roles in HCV life cycle [37–45]. Downregulation of viral replications was assessed by semiquantitative RT-PCR, western blotting, and immunostaining.

## 2. Materials and Methods

### 2.1. Cell Culture

Huh-7.5 cells (a kind gift from Dr. V. N. Pandey, New Jersey Medical School, USA), a highly permissive cell line for HCV replication, were grown in Dulbecco's Modified Eagle's Medium (Gibco, USA) supplemented with 10% FBS (Gibco, USA), penicillin 100 U/mL and streptomycin 100 *μ*g/mL (HiMedia, India), 0.1 mM nonessential amino acids (Gibco, USA), and 2 mM glutamax-I (Gibco, USA). Cells were maintained in a humidified incubator at 37°C in 5% CO_2_.

### 2.2. HCV Production in Cell Culture

For HCV culture, pFL-J6/JFH plasmid [24] (a kind gift from Dr. V. N. Pandey, New Jersey Medical School, USA) encoding full-length chimeric HCV genotype 2a was linearized with XbaI and subjected to* in vitro* transcription using MEGAscript Kit (Ambion, USA) following manufacturer's protocol. Huh-7.5 cells were transfected with 10 *μ*g transcript using Lipofectamine 2000 transfection reagent (Invitrogen, USA) using manufacturer's protocol and 6 h after transfection media were removed, cells were washed with PBS and incubated in fresh media. The culture supernatant was collected after 72 h and passed through 0.45 *μ*m filter and stored at −80°C in small aliquots.

### 2.3. Viral Load Quantification

Viral RNAs were isolated from cell culture supernatants using Trizol LS reagent (Ambion, USA) and quantified by real-time quantitative RT-PCR using primer pair of HCV 5′UTR: forward, 5′-CCTAATAGGGGCGACACTCCG-3′; reverse, 5′-CCACAAGGCCTTTCGCAACC-3′. Assays were performed using Sybr green real-time PCR master mix reagent kit (Invitrogen, USA) and mastercycler ep realplex2 (Eppendorf, Germany) according to the manufacturer's instructions.

### 2.4. RNA Isolation and RT-PCR

Total RNA was extracted from cells using Trizol reagent (Ambion, USA) following manufacturer's guidelines. RNA was then subjected to cDNA synthesis using RevertAid reverse transcriptase (Thermo Fisher Scientific, USA) following manufacturer's protocol in a thermocycler (Eppendorf, Germany). In brief, cDNA was synthesized using 2 *μ*g of total RNA in 20 *μ*L reaction volume using gene specific reverse primers and further 2 *μ*L of cDNA was used for PCR amplification of specific genes. Respective primer sequences were La autoantigen: forward, 5′-GGATAGACTTCGTCAGAGGAGCA-3′; reverse, 5′-CTGGTCTCCAGCACCATTTTCTG-3′; hVAP-A: forward, 5′-GTGCTCCATCTGATTTACCCCA-3′; reverse, 5′-TTCCACAGGCTTGCTCAGTATT-3′; PSMA7: forward, 5′-GCCATCACCGTCTTCTCGCC-3′; reverse, 5′-CGTTGTCATCCAAAGCACAG-3′; HCV NS5B: forward, 5′-ACATCAAGTCCGTGTGGAAGG-3′; reverse, 5′-GCTCCCATTACCGCCTGAGG-3′; and *β*-actin: forward, 5′-GCGGGAAATCGTGCGTGACATT-3′; reverse, 5′-GATGGAGTTGAAGGTAGTTTCGTG-3′. The PCR products were resolved by electrophoresis in 1.5% (w/v) agarose gels and images were captured by a Chemidoc XRS system (Bio-Rad, USA). The band intensities were quantified using Quantity One software.

### 2.5. Design and Synthesis of siRNAs

siRNAs were designed using IDT online designing tool and were synthesized from IDT, USA. Sequences of siRNAs were as follows: La autoantigen (GenBank accession number NM_003142): sense, 5′-GGAACAAAGAAGUGACUU-3′; antisense, 5′-ACUUCCCAAGUCACUUCU-3′; hVAP-A (GenBank accession number NM_194434.2): sense, 5′-GGUUUAGACAGGUUCAAUUAGCUCA-3′; antisense, 5′-UGAGCAAAUUGAACCUGUCUAAACCCG-3′; PSMA7 (GenBank accession number NM_002792.3): sense, 5′-CCUCUUCCAAGUGGAGUA-3′; antisense, 5′-CUGCGCGUACUCCACUUG-3′; HCV NS5B (HCV genotype 2a strain): sense, 5′-CCCUCUAUGACAUUACACA-3′; antisense, 5′-UGUGUAAUGUCAUAGAGGG-3′; scrambled negative control: sense, 5′-CUUCCUCUCUUUCUCUCCCUUGUGA-3′; antisense, 5′-UCACAAGGGAGAGAAAGAGAGGAAGGA-3′.

### 2.6. siRNA Transfection and HCV Infection

Huh-7.5 cells (2 × 10^5^ cells/well) grown overnight in 12-well plates were transiently transfected with 50 nM for single and 100 nM for combinations of siRNAs using Lipofectamine RNAiMAX transfection reagent (Invitrogen, USA) as per manufacturer's protocol. After 48 h of transfection, cells were infected with an equal amount of HCV (MOI 1 : 0.1) and incubated for 4 h. Cells were once again transfected with siRNAs to maintain the gene silencing effect and incubated for another 48 h. Total RNA or protein was isolated and subjected to RT-PCR or western blotting, respectively. Scrambled siRNA, noncomplementary to any known sequence in the human and HCV genome, was included as a negative control.

### 2.7. Western Blotting

For western blotting, cells were lysed with RIPA buffer (50 mM Tris-Cl, 5 mM EDTA, 150 mM NaCl, 1% Triton X-100, 1% sodium deoxycholate, and 1% sodium dodecyl sulfate) supplemented with 1 mM protease inhibitor, PMSF, and then centrifuged at 13,000 rpm for 10 min at 4°C. Supernatants were collected and total protein was quantified by Bradford method. Equal amount of protein (20 *μ*g) from the control and transfected cells was subjected to SDS-PAGE and then transferred to a PVDF membrane (GE healthcare, USA). After blocking with 5% BSA, blots were incubated overnight at 4°C with primary antibodies specific for La autoantigen (ab75927, Abcam, UK), HCV NS5B (sc-34040, Santa Cruz Biotechnology, USA), and *β*-actin (NB100-56874, Novus Biologicals, USA). Membranes were incubated with corresponding secondary antibodies conjugated with horseradish peroxidase (sc-2005, sc-2354, and sc-2004, Santa Cruz Biotechnology, USA). The blots were developed using ECL chemiluminescence detection kit (Bio-Rad, USA) by a Chemidoc XRS system (Bio-Rad, USA) and band intensities were quantified using Quantity One software [46].

### 2.8. Indirect Immunofluorescence

Huh-7.5 cells (5 × 10^4^ cells/well) grown on 17 mm poly-D-lysine coated glass coverslips (MP Biomedicals, USA) were fixed with 4% paraformaldehyde for 20 min at room temperature. Cells were washed twice with PBS and then permeabilized with 0.2% Triton–X100 for 10 min followed by blocking with 3% BSA in PBS. After blocking, cells were incubated overnight at 4°C with primary antibody specific for HCV NS5B (sc-34040, Santa Cruz Biotechnology, USA). Cells were then incubated with FITC conjugated secondary antibody (sc-2356, Santa Cruz Biotechnology, USA) for one hour at room temperature and then mounted the coverslips on glass slides with mounting media (50 mM Tris pH 8, 150 mM NaCl, and 1 mg/mL p-phenylenediamine in 50% glycerol) containing 400 nM DAPI and observed under a confocal laser scanning microscope (Olympus Fluoview FV1000) [47].

### 2.9. Cellular Viability Assay

The cytotoxicity of siRNA-Lipofectamine RNAiMAX complex was assessed by MTT [3-(4,5-dimethylthiazol-2-yl)-2,5-diphenyltetrazolium bromide] assay [48]. Huh-7.5 cells grown overnight in 96-well plate (20,000 cells/well) were transfected with individual or combination of siRNAs and incubated for 72 h. After incubation, 20 *μ*L of 5 mg/mL MTT reagent (HiMedia Laboratories, India) was added to each well and incubated for 4 h at 37°C. The formazan crystals were dissolved in acidified isopropanol solution and absorbance was taken in a microplate reader (iMark Microplate Reader, Bio-Rad, USA) at 595 nm with a reference wavelength of 650 nm.

### 2.10. Statistical Analysis

Data are presented as mean ± standard deviation (*n* = 3) and one-way ANOVA test was performed using SPSS software (version 22.0.0.0). *P* value < 0.05 versus control was considered statistically significant.

## 3. Result

### 3.1. Downregulation of Cellular and Viral Genes Severely Inhibits HCV Replication

HCV NS5B, an RNA-dependent RNA polymerase, synthesizes viral genome through an intermediate negative RNA strand. Hence, it is an important target to inhibit the viral replication. Among the host cell factors, La autoantigen, an RNA-binding protein, plays an important role in viral translation and replication and protects viral RNA from rapid degradation [37–39]. Expression of La autoantigen is induced by HCV infection, and it activates telomerase activity in Huh-7.5 cells. Therefore, it could be important to suppress La autoantigen protein not only for the inhibition of HCV, but also for reducing the oncogenic potential of hepatocytes infected with HCV [49]. PSMA7, *α*-subunits of 20S proteasome, regulates HCV-IRES (internal ribosome entry site) mediated cap-independent viral translation, a phenomenon essential for HCV replication. Furthermore, inhibition of the 20S proteasome by proteasome inhibitor exerts a dose-dependent inhibition on HCV-IRES activity [40]. Another host cell factor, hVAP-A, a SNARE-like vesicle transport protein, is present in the ER and ER/Golgi intermediate compartment (ERGIC) and plays a general role in vesicle traffic between the ER and Golgi compartment [50]. It makes a bridge between viral proteins NS5B and NS5A by making its N-terminus interact with NS5B and C-terminus interact with NS5A and mediates the formation of HCV replication complex on a lipid raft [41]. We downregulated these viral and host genes using single and combinatorial RNAi-based approach in Huh-7.5 cells infected with HCV and evaluated its effect on viral replication.

**Figure 1:**
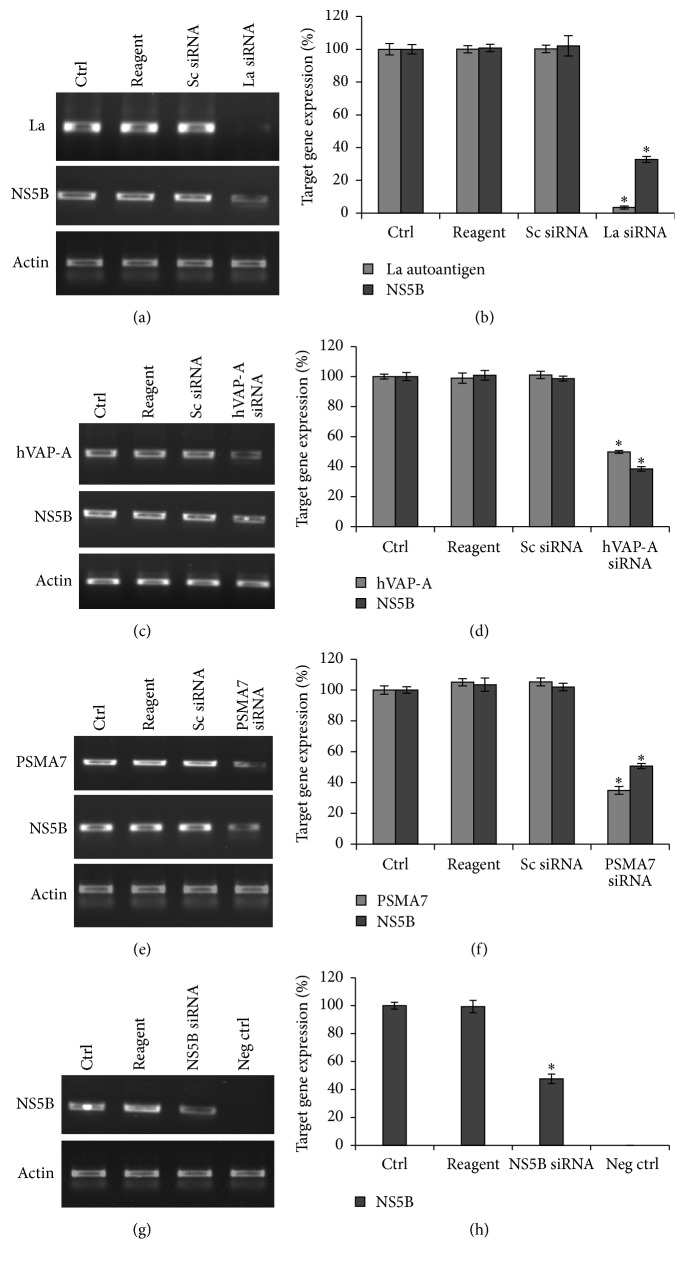
Downregulation of La autoantigen, hVAP-A, PSMA7, and NS5B leads to reduction in viral replication: overnight grown Huh-7.5 cells were transfected with La autoantigen, hVAP-A, and PSMA7 siRNAs for 48 h. Cells were then infected with the virus and again transfected with same siRNAs. After incubation total RNA was isolated and subjected to RT-PCR. NS5B siRNA was transfected once only after cells were infected. (a), (c), (e), and (g) represent RT-PCR results for target genes and NS5B gene upon treatment with La autoantigen, hVAP-A, PSMA7, and NS5B siRNAs, respectively. Densitometric analysis of (a), (c), (e), and (g) images is expressed in percentage in (b), (d), (f), and (h), respectively. The data represent mean ± standard deviation. ^*∗*^ *P* value < 0.05 versus control considered statistically significant.

We found that siRNA targeted against La autoantigen mRNA reduced its expression by 96.5% in HCV-infected Huh-7.5 cells and consequently HCV replication was also reduced by 67.2% (Figures 1(a) and 1(b)) indicating a direct impact of downregulation of La autoantigen on viral replication. Similarly, there was 39% reduction in viral replication upon 50.1% downregulation of hVAP-A (Figures 1(c) and 1(d)) and 65% downregulation of PSMA7 resulted in 50.7% lower level of HCV level (Figures 1(e) and 1(f)). These observations clearly indicate a reduction in HCV replication in response to downregulation of selected cellular genes, involved in viral life cycle. Similarly, HCV propagation was also inhibited by 52% compared to control upon treatment with siRNA targeted against NS5B region of HCV genome (Figures 1(g) and 1(h)).

### 3.2. Effects of Combinatorial Gene Silencing on HCV Replication

**Figure 2 fig2:**
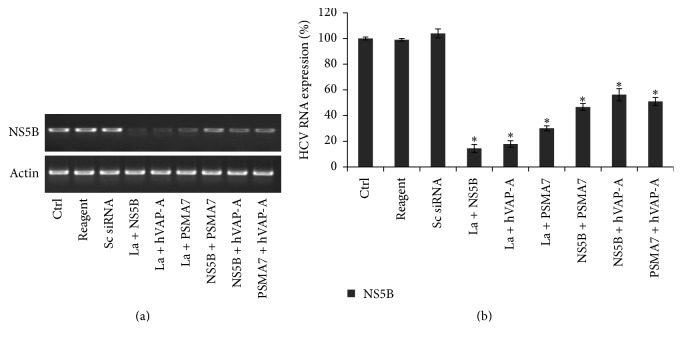
Simultaneous downregulation of multiple genes more effectively inhibits HCV replication: Huh-7.5 cells in a similar experimental setup as mentioned in Figure 1 were transfected with following combinations of siRNAs: La autoantigen + NS5B, La autoantigen + hVAP-A, La autoantigen + PSMA7, NS5B + hVAP-A, NS5B + PSMA7, and PSMA7 + hVAP-A. Downregulation of viral replication were determined by RT-PCR as shown in (a). (b) represents densitometry analysis of HCV NS5B downregulation. The data represent mean ± standard deviation. ^*∗*^ *P* value < 0.05 versus control was considered statistically significant.

In order to determine the effect of simultaneous downregulation of multiple genes on HCV replication, Huh-7.5 cells were transfected with the following combinations of siRNAs: La autoantigen + NS5B, La autoantigen + hVAP-A, La autoantigen + PSMA7, NS5B + PSMA7, NS5B + hVAP-A, and PSMA7 + hVAP-A. Viral replication was reduced by 85.6%, 82.2%, 69.9%, 43.8%, 53.3%, and 49.0% with combinations of La autoantigen + NS5B, La autoantigen + hVAP-A, La autoantigen + PSMA7, NS5B + PSMA7, NS5B + hVAP-A, and PSMA7 + hVAP-A siRNAs, respectively (Figure 2). The combination of La autoantigen + hVAP-A siRNAs reduced the HCV replication by 82.2% while it was 67.2% and 39%, respectively, when the genes were downregulated individually (Figure 1). Similarly, the combination of La autoantigen + NS5B siRNAs also inhibited the HCV replication by 85.6%, whereas the individual downregulation of La autoantigen and NS5B genes demonstrated 67.2% and 52% inhibition of viral replication, respectively (Figure 1). These results indicate synergistic anti-HCV effects due to simultaneous downregulation of La autoantigen + hVAP-A or La autoantigen + NS5B on viral replication. However, similar synergistic effects on viral replication were not observed after simultaneous downregulation of La autoantigen + PSMA7, NS5B + PSMA7, NS5B + hVAP-A, or PSMA7 + hVAP-A siRNAs. This indicates that multiplexing any combination of siRNAs is not sufficient to have a synergistic antiviral effect (Table 1). Their roles in viral biology are crucial to be effective antiviral targets.

### 3.3. Combinatorial Effect of La Autoantigen with NS5B siRNA on HCV Protein Expression

**Figure 3:**
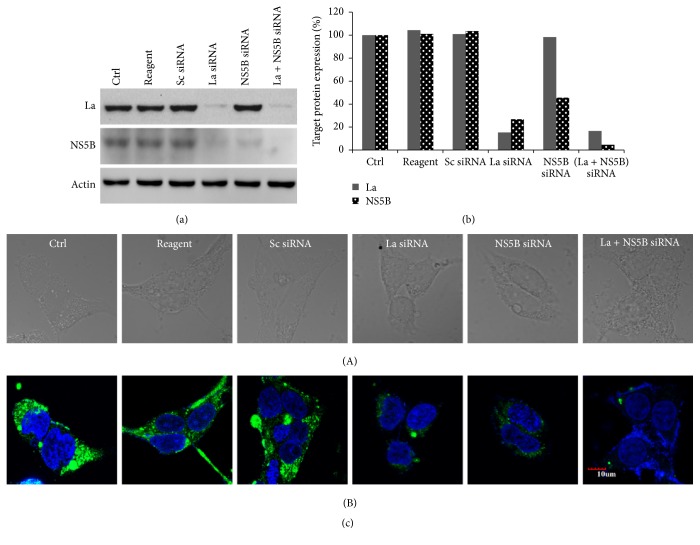
Analysis of NS5B protein expression after transfecting NS5B and La autoantigen siRNAs: HCV-infected Huh-7.5 cells were treated with siRNAs against La autoantigen and NS5B as mentioned above. After 48 h total cellular protein was isolated and subjected to western blot analysis. (a) indicates downregulation of La autoantigen (upper gel) and NS5B (middle gel) and *β*-actin (lower gel). (b) represents densitometric analysis of La autoantigen and HCV NS5B expressed in percentage. Immunofluorescence of NS5B from single and combination of siRNAs transfected cells as represented in (c) where row (A) indicates cells in bright field and row (B) is the merging of DAPI and FITC stained cells.

**Figure 4:**
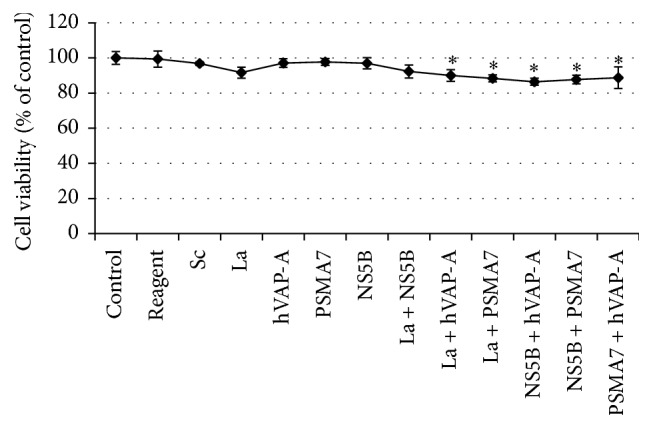
Cytotoxicity assay of single and different combinations of siRNAs: Huh-7.5 cells grown for 24 h in a 96-well plate were transiently transfected with single or different combination of siRNAs and incubated for 72 h. Cell viability was then determined by MTT assay. Absorbance was taken at 595 nm by a microplate reader and readings were plotted as percentage. The data represent mean ± standard deviation. ^*∗*^ *P* value < 0.05 versus control was considered statistically significant.

We further checked the inhibitory effect of downregulation of La autoantigen and HCV NS5B on the expression of the viral protein by western blot analysis. We found that combination of La autoantigen + NS5B siRNA demonstrated 95% reduction in NS5B protein levels compared with 73% and 54.5%, respectively, when the genes were downregulated individually (Figures 3(a) and 3(b)). Similar results were obtained from immunostaining of NS5B protein. As shown in Figure 3(c), downregulation of the viral protein NS5B was observed and it was found that cells treated with combination of siRNAs showed a cumulative effect on HCV inhibition compared to individual siRNAs treated cells. Cytotoxicity for all the siRNAs was measured at 50 nM (for single siRNAs) and 100 nM (for combinations of siRNAs) and no significant effect was observed on cell growth and viability upon treatment with test or control siRNAs (Figure 4).

## 4. Discussion

**Figure 5:**
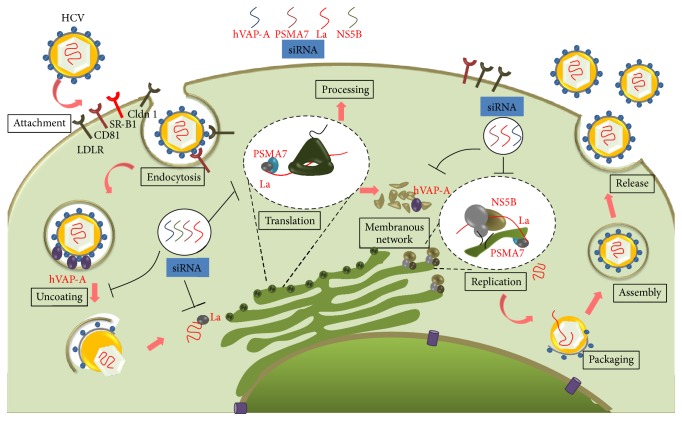
Schematic model of HCV life cycle and siRNA-mediated inhibition of different steps: After receptor-mediated entry, host protein hVAP-A interacts in the uncoating and replication complex formation step within the membranous web network region. Therefore, downregulating hVAP-A with siRNA severely affects the viral replication. siRNA against La autoantigen and PSMA7 simultaneously hinder viral translation and replication step. La autoantigen also binds to the 3′UTR of viral RNA and protects it from cellular nuclease; hence downregulation of La autoantigen makes the viral RNA susceptible to nuclease. NS5B siRNA was directed against the NS5B conserved region of viral RNA.

The HCV life cycle is broadly divided into following major steps: entry and uncoating, translation, replication, assembly, and release of virion particles, as depicted in Figure 5 [51, 52]. Each of these steps involves interactions with the host cell factors which are crucial to establish infection and maintain the viral life cycle [53, 54]. Interventions of these interactions can severely impair the viral life cycle. Cellular factors hVAP-A, La autoantigen, and PSMA7 have multiple roles during distinct stages of HCV life cycle and are potential targets for host targeting antivirals (HTA) as depicted in the schematic model of HCV life cycle in Figure 5 [37–43, 55, 56].

During uncoating, the internalized viral nucleocapsid fuses with endosomal membranes and release its genome into the cytosol [57]. Alteration in cholesterol composition in the endosomal compartment can inhibit this step. hVAP-A with oxysterol-binding protein (OSBP) is involved in cholesterol homeostasis which in turn helps endocytic release of virion into the cytosol [42, 43]. Therefore, downregulation of hVAP-A inhibits the fusion of clathrin coated virion with endosomal membranes, thereby inhibiting then invasion step. In the present study upon 50.1% downregulation of hVAP-A, HCV was also inhibited by 39% (Figures 1(c) and 1(d)). However, upon more efficient downregulation of hVAP-A ~60% reduction in HCV replication has also been observed [58]. Short hairpin against hVAP-A expressed through an adenoviral vector effectively inhibits the HCV replication [59]. Variations in the level of downregulation of target gene and HCV could be attributed to the silencing efficiency of siRNA as well as different HCV strains.

Following entry into the cytosol, the viral RNA becomes highly susceptible to siRNA-mediated degradation. A number of groups have shown that NS5B is an important factor for HCV replication and an attractive antiviral drug target [60, 61]. We designed siRNA targeting the active site of HCV NS5B, a highly conserved region among different HCV genotypes. As expected, upon downregulation of NS5B gene significant decrease in viral RNA and protein expression was observed (Figures 1(g), 1(h) [Fig fig2], and 3). Several groups have directed siRNAs against different regions of viral genome including NS5B and demonstrated different levels of inhibition of viral replication and protein expression [62, 63]. During HCV life cycle several cellular factors bind to the viral RNA at different regions in order to help with translation and replication or to protect viral RNA [26, 37–39, 64]. The cellular factor La autoantigen binds to the U-rich region of HCV 3′UTR and shield viral RNA from degradation [39]. Therefore, lower expression of La autoantigen makes the viral RNA vulnerable to cellular nucleases.

HCV uses host translation machinery and several cellular factors to synthesize polyprotein precursor. La autoantigen also binds to the initiator AUG codon of 5′UTR and facilitates efficient HCV-IRES mediated translation [38]. In our study when La autoantigen was downregulated by 96.5%, consequently reduction of 67.2% in HCV level was also observed (Figures 1(a) and 1(b)). In few studies, La autoantigen was used as a therapeutic target for successful inhibition of HCV, although the extent of inhibition of target gene and HCV varied among different studies due to different siRNA target regions, experimental setup, or efficiency of siRNA delivery [58, 59]. PSMA7 has been implicated for its role in IRES mediated translation of HCV [40]. We downregulated PSMA7 by 65% and as a result of this viral replication was also reduced by 50.7% (Figures 1(e) and 1(f)). About 50% inhibition of HCV replication has also been observed when PSMA7 expression was downregulated by the siRNA delivered through retroviral vector [36].

HCV makes a replication complex within cholesterol-rich lipid raft membrane using viral nonstructural proteins, NS3, NS4A, NS4B, NS5A, and NS5B, and cellular protein hVAP-A. The cellular factor makes a bridge between viral proteins NS5B and NS5A [41]. Therefore, due to downregulation of hVAP-A the association of HCV nonstructural proteins on lipid raft membrane is severely disturbed which consequently hampers viral replication. Several cellular factors and HCV nonstructural proteins are involved during viral replication [65, 66]. La autoantigen and PSMA7 have been involved at this stage and hence downregulation of these genes severely affected the viral replication (Figures 1(a), 1(b), 1(e), and 1(f)). Therefore, these cellular proteins are the important anti-HCV target. The overall schematic model of HCV life cycle and siRNA-mediated inhibition at different steps has been shown in Figure 5.

It is also important to confirm that silencing of these cellular genes has no lethal effects on the host. La autoantigen influences RNA polymerase III transcription* in vitro* [67], but depletion does not hamper the transcription rate synthesized by class III RNA polymerase although reduction in ribonucleoprotein (RNP) formation has been observed [68]. hVAP-A is predominantly involved in vesicle trafficking between membrane compartments including endoplasmic reticulum and Golgi complex [69, 70] and reduction of hVAP-A may affect the vesicle transport functions of cells. siRNA-mediated inhibition of PSMA7 reduced vesicular stomatitis viral replication through enhanced production of virus-induced type I IFN [71]; still it may be speculated that downregulation may hamper the intracellular proteolysis. However, MTT assay on cells transfected with all the siRNAs individually or in combination showed no significant changes in cellular viability compared with the nontransfected controls (Figure 4).

When multiple genes were downregulated and more than one viral step was challenged, inhibition of HCV was more pronounced (Table 1). Viral replication was reduced by 85.6% and 82.2% particularly with combinations of siRNAs against La autoantigen with NS5B or hVAP-A, respectively, as compared to other combinations of siRNAs (Figure 2). These observations confirmed that downregulation of La autoantigen along with NS5B or hVAP-A facilitates inhibition of HCV replication cooperatively. These results were also confirmed by western blotting and immunostaining (Figure 3(c)). The combination of La autoantigen with NS5B simultaneously targets the viral RNA, translation, and replication steps. Similarly, when La autoantigen and hVAP-A were simultaneously downregulated viral uncoating, translation, replication complex formation, replication, and viral RNA were concurrently affected. A few recent studies exhibited the effect of combinatorial RNAi on HCV replication. Enhanced reduction in HCV entry was noted after downregulation of entry receptors CD81 along with LDLR or SR-BI [33]. In another study, dual siRNAs directed against HCV 5′UTR rapidly inhibit viral replication and also minimized escape mutants [34]. Adeno-associated viral (AAV) vector expressed multiple short hairpin oligos against HCV NS5B and HCV 5′UTR has shown long lasting HCV inhibition in a nonhuman primate model [35]. siRNAs against HCV envelope protein E2 along with entry receptor LDLR or CD81 showed an enhanced reduction in HCV entry [32]. However, these studies were targeted to either viral entry or the viral genome. Virus is often able to escape the single inhibitory blocks created by antivirals and capable of perpetuating its life cycle. Therefore, targeting multiple steps at the same time is more realistic approach in order to inhibit HCV more effectively and prevent escape mutants. It is pertinent to mention that designing of siRNA and selection of viral and cellar targets are the key to the success of siRNA based inhibition of HCV. Application of siRNA has tremendous possibilities because it not just allows downregulating host and viral factors simultaneously but also allows tremendous flexibility in designing of siRNAs with dynamic changes in the viral sequences.

## 5. Conclusion

Currently approved anti-HCV drugs are mainly genotype dependent and because of error prone replication of HCV emergence of quasi-species are inevitable. Targeting conserved regions among different genotypes can reduce emergence of resistant strains and also inhibit virus in a pan-genotypic manner. Targeting host cell factor is a novel approach particularly for patients who fail to respond to current direct antiviral agents (DAA) treatment or develop drug resistance. As siRNAs work at mRNA level the host DNA remains unaffected and emergence of mutational resistance to the host or the virus does not arise, therefore, creating a possibility of pan-genotypic anti-HCV treatment. Several studies showed that siRNAs targeted against different regions (stem-loops II, III, and IV) of 5′UTR or cellular factor CD81 inhibit HCV infection in genotype-independent manner [72–74]. Therefore, siRNA has tremendous potential as a therapeutic tool against HCV. We showed simultaneous inhibition of La autoantigen with NS5B or hVAP-A is more effective than silencing a single gene to inhibit the viral replication. Therefore, a cocktail of siRNAs directed against different crucial steps in viral life cycle is likely to be a more effective approach in the treatment of chronic hepatitis C.

## Figures and Tables

**Table 1:** Effect of siRNAs used individually and in combination on HCV replication. Only combination of La autoantigen with hVAP-A or HCV NS5B shows syndergistic effect on HCV inhibition. Other combinations of siRNAs could not improve the overall level of inhibition of HCV.

Target gene (siRNA position)	Target gene silencing (%)	Reduction of HCV after single gene silencing (%)	Reduction of HCV after multiple gene silencing (%)			
			(+) La autoantigen	(+) hVAP-A	(+) PSMA7	(+) NS5B
La autoantigen (1064–1081)	96.5	67.2	—	—	—	—
hVAP-A (5929–5953)	50.1	39.0	82.2↑	—	—	—
PSMA7 (199–217)	65.0	50.7	69.9↑	49.0↓	—	—
HCV NS5B (531–549)	52.0	52.0	85.6↑	53.3↓	43.8↓	—
